# Comparison of Two Commercial Colorimetric Broth Microdilution Tests for *Candida* Susceptibility Testing: Sensititre YeastOne versus MICRONAUT-AM

**DOI:** 10.3390/jof7050356

**Published:** 2021-05-01

**Authors:** Sophie Philips, Frederik Van Hoecke, Emmanuel De Laere, Steven Vervaeke, Roos De Smedt, Jerina Boelens, Deborah De Geyter, Denis Piérard, Katrien Lagrou

**Affiliations:** 1 Department of Laboratory Medicine, AZ Delta Hospital, Deltalaan 1, 8800 Roeselare, Belgium; Frederik.VanHoecke@azdelta.be (F.V.H.); Emmanuel.DeLaere@azdelta.be (E.D.L.); Steven.Vervaeke@azdelta.be (S.V.); roos.desmedt@azdelta.be (R.D.S.); 2 Department of Microbiology and Infection Control, Vrije Universiteit Brussel (VUB), Universitair Ziekenhuis Brussel (UZ Brussel), Laarbeeklaan 101, 1090 Brussels, Belgium; Deborah.DeGeyter@uzbrussel.be (D.D.G.); Denis.Pierard@uzbrussel.be (D.P.); 3 Department of Laboratory Medicine, Ghent University Hospital, Corneel Heymanslaan 10, 9000 Ghent, Belgium; Jerina.Boelens@uzgent.be; 4 Department of Laboratory Medicine and National Reference Centre for Mycosis, University Hospitals Leuven, Herestraat 49, 3000 Leuven, Belgium; katrien.lagrou@uzleuven.be

**Keywords:** *Candida*, antifungal agents, antifungal susceptibility testing, colorimetry, CLSI, EUCAST, MICRONAUT-AM, Sensititre YeastOne, MIC

## Abstract

Two colorimetric broth microdilution antifungal susceptibility tests were compared, Sensititre YeastOne and MICRONAUT-AM for nine antifungal agents. One hundred clinical *Candida* isolates were tested, representing a realistic population for susceptibility testing in daily practice. The reproducibility characteristics were comparable. Only for fluconazole, caspofungin, 5-flucytosine and amphotericin B, an essential agreement of ≥90% could be demonstrated. Sensititre minimal inhibitory concentrations (MICs) were systematically higher than MICRONAUT MICs for all antifungals, except for itraconazole. CLSI clinical breakpoints (CBPs) and epidemiological cut-off values (ECVs) were used for Sensititre MICs while for MICRONAUT the EUCAST CBPs and ECVs were used. Only fluconazole, micafungin, and amphotericin B had a categorical agreement of ≥90%. For fluconazole, micafungin, and amphotericin B the susceptibility proportions were comparable. Susceptibility proportion of posaconazole and voriconazole was higher using the MICRONAUT system. For itraconazole and anidulafungin, the susceptibility proportion was higher using Sensititre. It was not possible to determine the true MIC values or the correctness of a S/I/R result since both commercial systems were validated against a different reference method. These findings show that there is a significant variability in susceptibility pattern and consequently on use of antifungals in daily practice, depending on the choice of commercial system.

## 1. Introduction

The number of opportunistic fungal infections has increased in recent years. These opportunistic pathogens can cause a wide range of infections ranging from mild dermatosis to serious systemic infections. *Candida* species are the most common cause of invasive fungal infections [1,2]. Candidemia is an important cause of morbidity and mortality in high-risk patient groups. The prevalence of *Candida* infections is high in immunocompromised patients, transplant patients, patients with cancer and patients with a prolonged stay at the intensive care unit. Other common risk factors include use of broad-spectrum antibiotics, abdominal surgery, indwelling central venous and urinary catheters, total parenteral nutrition and hemodialysis [1,3]. Compared to antibiotics, the number of antifungal agents is rather limited [4]. The antifungal drug classes comprise azoles, echinocandins, polyenes and 5-flucytosine [5]. Different *Candida* species exhibit diverse susceptibility profiles towards antifungal agents and various trajectories to acquire resistance when exposed to antifungals. In bacteria, resistance usually involves the transfer of mobile genetic elements between strains or species, while *Candida* yeasts have a high genomic plasticity and resistance is usually based on genetic alterations. Mechanisms of acquired resistance can be classified in mutations that increase the expression of the target or the alteration of its binding affinity towards the antifungal agent and mutations which reduce the intracellular accumulation of the drug [6]. The increased use of antifungal prophylaxis and empirical treatment in high-risk populations for candidiasis gave rise to fungal organisms with decreased susceptibility or resistance and in some cases, cross-resistance exists between antifungal agents [7,8]. Because of these increasing numbers of multi-drug resistant organisms, a reliable, reproducible, and clinically relevant antifungal susceptibility test (AFST) is crucial to guide antifungal therapy [7,9,10]. Both EUCAST (European Committee on Antimicrobial Susceptibility Testing) and CLSI (Clinical and Laboratory Standards Institute) have a reference broth microdilution method for AFST available [11]. However, these reference methods are time-consuming, labor-intensive and too complex to be deployed in a routine clinical laboratory setting and commercial assays have become available to overcome these practical concerns. The commercial methods offer a fast, simple, flexible, easy-to-perform, more practical and time-saving alternative [7,11,12,13]. The Sensititre YeastOne (Thermo Fisher Scientific, Waltham, MA, USA) and the MICRONAUT-AM (MERLIN Diagnostika GmbH, Bornheim, Germany) AFST are two commercial colorimetric broth microdilution tests. 

The objective of this study was to compare Sensititre YeastOne, which is further indicated in the text as “SY”, with MICRONAUT-AM, which is further indicated in the text as “M-AM”. The *Candida* collection used in this study represents a realistic population of *Candida* isolates for which susceptibility testing has to be carried out by the routine laboratory. Consequently, the results of this study are predictive for the impact a routine clinical laboratory will experience when choosing one or the other assay.

## 2. Materials and Methods

**Isolates.** One hundred clinical isolates of *Candida* species were tested, including 26 *Candida glabrata*, 13 *Candida albicans*, 12 *Candida parapsilosis*, 10 *Candida tropicalis*, 8 *Candida lusitaniae*, 7 *Candida dubliniensis*, 5 *Candida krusei*, 5 *Candida inconspicua*, 4 *Candida guilliermondii*, 2 *Candida rugosa*, 1 *Candida kefyr*, 1 *Candida pararugosa*, 1 *Candida lambica*, 1 *Candida auris*, 1 *Candida utilis*, 1 *Candida pulcherrima*, 1 *Candida allociferrii*, and 1 *Candida nivariensis*. The identification of the isolates was performed with matrix-assisted laser desorption ionization time-of-flight mass spectrometry (MALDI-TOF MS). Most isolates were recovered from blood. The remainder were recovered from the respiratory tract, abdomen, vagina, wounds and urine. One *C. guilliermondii* was isolated from a toenail and the *C. auris* was a quality control strain provided by the Belgian national public health institute. The *Candida* isolates were obtained from the culture collections of four Belgian hospitals (AZ Delta Roeselare, University Hospitals Leuven, Ghent University Hospital and University Hospital Brussels). A broad range of MICs are represented by the isolates and some azole and echinocandin resistant strains were included. With each test run, two American Type Culture Collection (ATCC) strains (ATCC 22019 *C. parapsilosis* and ATCC 6258 *C. krusei*) were included as quality control [14,15,16]. 

**Sensititre YeastOne AFST.** The antifungals included in this test are anidulafungin (0.015–8 mg/L), micafungin (0.008–8 mg/L), caspofungin (0.008–8 mg/L), fluconazole (0.12–256 mg/L), posaconazole (0.008–8 mg/L), voriconazole (0.008–8 mg/L), itraconazole (0.015–16 mg/L), amphotericin B (0.12–8 mg/L), and 5-flucytosine (0.06–64 mg/L). All *Candida* isolates were grown on a Sabouraud Glucose Agar plate (Thermo Fisher Scientific™, Waltham, MA, USA) and the test was further performed according to the procedure described by the manufacturer [14]. 

**MICRONAUT-AM AFST.** The antifungals included in this test are anidulafungin (0.002 mg/L, 0.015–8 mg/L), micafungin (0.002 mg/L, 0.015–8 mg/L), caspofungin (0.002 mg/L, 0.015–8 mg/L), fluconazole (0.002 mg/L, 0.25–128 mg/L), posaconazole (0.0078–8 mg/L), voriconazole (0.0078–8 mg/L), itraconazole (0.031–4 mg/L), amphotericin B (0.031–16 mg/L), and 5-flucytosine (0.0625–32 mg/L). Several well-isolated *Candida* colonies were picked from the same pure 24-h culture of the *Candida* isolate grown on Sabouraud Glucose Agar as for the SY susceptibility test and the test was further performed according to the manufacturer’s procedure [15]. 

**Visual reading.** Both manufacturers make use of the same growth indicator, resazurin, which is an oxidation-reduction indicator. With sufficient fungal metabolism and growth, the oxidized, blue indicator switches to the reduced state, which has a pink color. It is possible that the produced resorufin (pink) undergoes further reduction to dihydroresorufin (white) due to intensive fungal growth [15,17,18,19]. Both test plates were read visually under normal laboratory lighting using a mirror. After 24 h of incubation, the positive growth control well was examined. When the color of the growth control had changed from blue to pink, the MIC values for the antifungal agents were read out. In case the color of the growth control was still blue or faintly purple, the test plates were re-incubated and re-examined after 36 h (M-AM) or after 48 h (SY). When the growth control of M-AM test plates was still blue or faintly purple after 36 h, the plates were reincubated and reexamined after 48 h. The MIC was read according to the recommendations of the manufacturers [14,15]. 

**Intralaboratory reproducibility.** For the intralaboratory reproducibility, two ATCC strains (ATCC 22019 *C. parapsilosis* and ATCC 6258 *C. krusei*) were tested on four different days. For each ATCC strain-antifungal agent combination the modal MIC was calculated. The reproducibility is defined as the percentage of MICs within the modal MIC ± one dilution. When the modal MIC consists of two adjacent dilutions for example 0.125 and 0.25 mg/L, the reproducibility is defined as the percentage of MICs within a 4-dilution range, so from 0.0625 to 0.5 mg/L for the example given [20].

**Essential agreement.** The MICs obtained with the SY method were compared to the MICs obtained with the M-AM method for the 100 clinical *Candida* isolates. The EA between those MICs was calculated for each antifungal agent. The EA is defined as the percentage of MICs within two dilutions between both methods [21]. 

**Comparison of MIC values.** The MIC values obtained with the M-AM panel were plotted against the MIC values obtained with the SY panel for each antifungal agent.

**Categorical agreement.** For each antifungal agent, the categorical agreement, defined as the agreement of interpretative results (S/I/R) between both methods, was calculated [21]. SY was developed and calibrated against the CLSI reference method [22], while M-AM was developed and calibrated against the EUCAST reference method [23]. As a consequence, CLSI clinical breakpoints (CBPs) or epidemiological cutoff values (ECVs) were used to assess the MICs obtained with SY and EUCAST CBPs or ECV were used to evaluate the MICs obtained with M-AM for determining the CA. When available, CBPs were used for classification of the *Candida* isolates as susceptible (S), intermediate (I), susceptible-dose dependent (SDD) or resistant (R). When isolates belong to the I or SDD category of CLSI and to the I category of EUCAST, they were considered as a categorical match in the CA calculation. If there were no CBPs, ECVs were used to classify the *Candida* isolates as wild-type (WT) isolates or non-wild-type (non-WT) isolates. For calculation of the CA, isolates that were classified as S with one method and WT with the other method were considered as a categorical match. Similar to this, isolates that were classified as R with one method and non-WT with the other method were considered as a categorical match too. When there were no CBPs nor ECVs, the *Candida* isolate could not be categorized and was not included in the calculation of the CA. There are no CLSI nor EUCAST CBPs or ECVs for 5-flucytosine. CLSI had CBPs for this molecule, but recent data suggested that the CBPs were not correct and should no longer be used [24]. Table 1 gives an overview of the CLSI and EUCAST CBPs and ECVs used in this study [24,25,26,27]. 

Subsequently, the number and percentage of CA discrepancies between SY and M-AM were calculated. CA discrepancies are subdivided into three types: minor discrepancies (mDs), major discrepancies (MDs), and very major discrepancies (VMDs). SY nor M-AM is a reference method, but in our case, SY was chosen as ‘reference method’ because SY was validated in a more robust way so far. Therefore the next partition was made: mD: I/SDD by one method and S/WT or R/non-WT by the other; MD: SY S/WT and M-AM R/non-WT; VMD: SY R/non-WT and M-AM S/WT. 

**Susceptibility ratios.** The percentage of S/WT and I/SDD *Candida* isolates for each antifungal agent, obtained with the M-AM system and the SY system was calculated and these results were presented visually. Chi-square test was performed to evaluate the differences to be statistically significant (*p* value of < 0.05).

## 3. Results

### 3.1. Intralaboratory Reproducibility

There was a high degree of reproducibility except for 5-flucytosine for the ATCC 6258 *C. krusei* strain tested with M-AM (Table 2). The observed MICs for this combination were 8 mg/L, 8 mg/L, 4 mg/L and 2 mg/L. Consequently, the modal MIC was 8 mg/L and the MIC of 2 mg/L was not within one dilution of the mode, resulting in a reproducibility of 75%. 

### 3.2. Essential Agreement

In Table 3 the EA as the percentage of MICs within two twofold dilutions between SY and M-AM is presented for the nine antifungal agents tested. The *C. lambica* isolate did not grow in the SY panel, nor in the M-AM panel, even after repeat testing although correct growth control was observed. So this strain was left out of further calculations.

Voriconazole and itraconazole showed a low EA of respectively 64.6% and 72.7%. Posaconazole had a very low EA of 38.3%. For fluconazole, however, an EA of 98.0% was demonstrated. The EA of echinocandins was 84.8% for micafungin, 89.9% for anidulafungin and 94.9% for caspofungin. For 5-flucytosine and amphotericin B, an EA of respectively 94.9% and 100% was noted. 

### 3.3. Comparison of MIC Values

**Figure 1:**
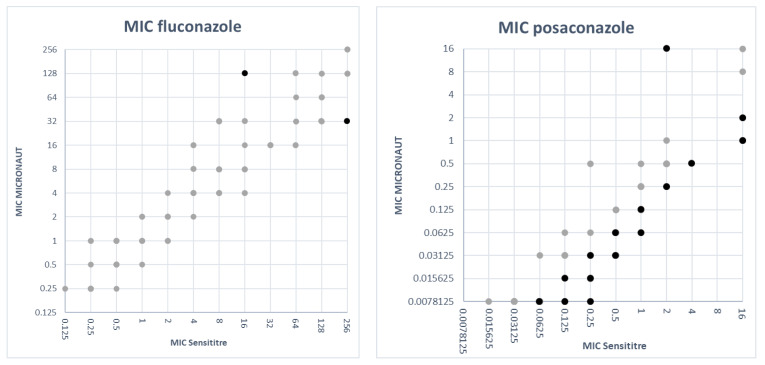

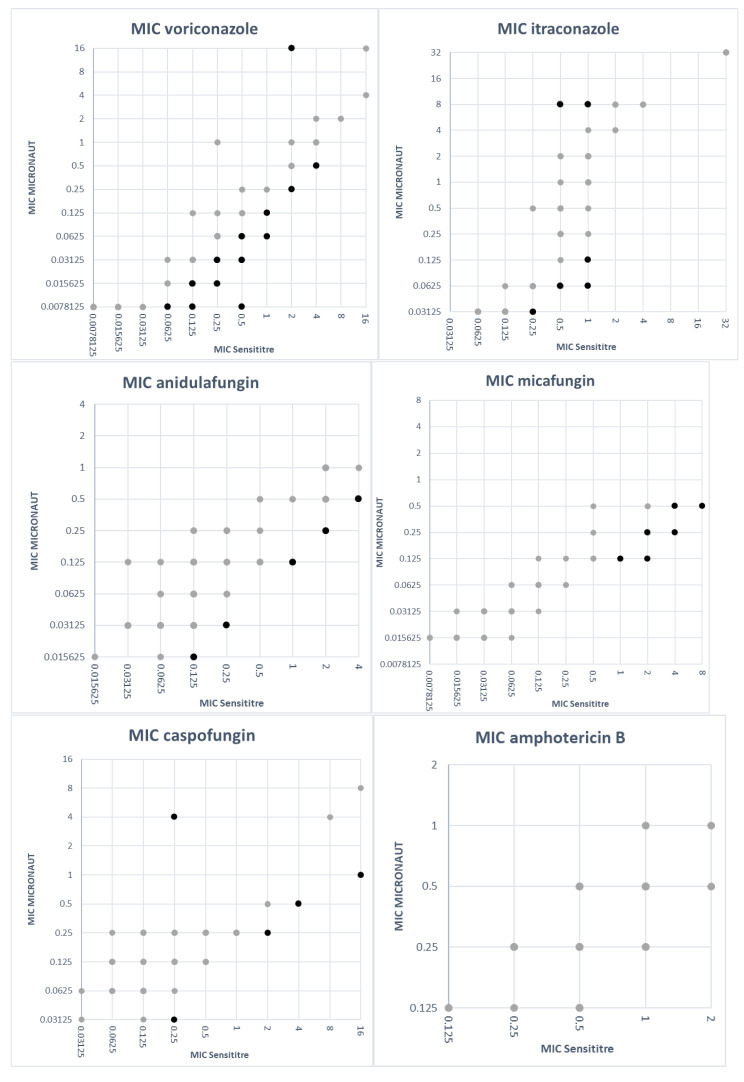

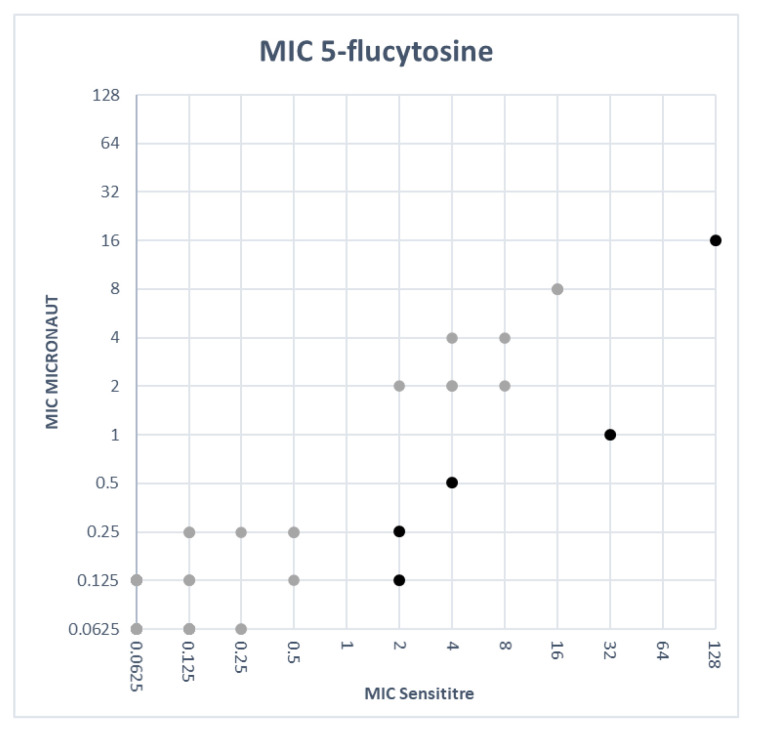
MIC values obtained with SY versus MIC values obtained with M-AM for each antifungal agent. The light colored dots represent MIC values within two dilutions between both methods. The dark colored dots represent MIC discrepancies of more than two dilutions.

In Figure 1 the MIC values obtained with the M-AM panel were plotted against the MIC values obtained with the SY panel for each antifungal agent. To create these plots, MIC values ‘≤X’ or ‘>X’ were adjusted as follows: ‘≤X’ was adjusted to ‘X’ (e.g., ≤0.125 was adjusted to 0.125) and ‘>X’ was set to the next twofold dilution (e.g., >4 was set to 8), except for >256, which was set to 256. For most antifungal agents the SY MICs were generally higher than the M-AM MICs (Figure 1). For itraconazole, the M-AM MICs were clearly higher than the SY MICs. Fluconazole MICs were very comparable between both methods with 44 isolates out of 99 having the same MIC value. Thirty-six isolates had a higher MIC with the SY system and 22 isolates showed higher MICs with the M-AM system. 

### 3.4. Categorical Agreement

The CA as well as the number and percentage of CA discrepancies between SY and M-AM are listed in Table 3. 

Because there are no CLSI and EUCAST CBPs nor ECVs for 5-flucytosine, the CA could not be calculated for this antifungal agent. 

As the *C. lambica* isolate did not grow in both panels, MIC results of only 99 isolates were available for CA determination. The CA for caspofungin could not be calculated because of susceptibility discordances between anidulafungin and micafungin. In these cases, EUCAST nor CLSI do provide a solution for determining the susceptibility of caspofungin as discussed further. 

For amphotericin B, 86 *Candida* isolates were included in the calculation of the CA. No discordances were found, which resulted in a CA of 100%. The remaining 13 isolates belonged to *Candida* species for which no CLSI or EUCAST CBPs or ECVs exist.

Non-species-related EUCAST CBPs (M-AM MICs) were used for *Candida* isolates without species-related breakpoints for fluconazole. The CA for fluconazole could be calculated on a total of 81 isolates. Because of *C. krusei*’s intrinsic resistance to fluconazole, the five *C. krusei* isolates were left out of the calculations and CLSI does not provide CBPs or ECVs for the remaining 13 isolates. A discordance was found for only 4 out of 81 isolates, resulting in a CA of 95.1%. There were three mDs which were due to a difference in MICs, 8 or 16 versus 32 mg/L (SY versus M-AM) for *C. glabrata*. One VMD was identified (2 versus 1 mg/L, SY versus M-AM) for a *C. lusitaniae* which resulted in a categorical interpretation of non-WT (CLSI) versus S (EUCAST). 

For posaconazole, 77 isolates were considered in the calculation of the CA, due to the unavailability of CBPs or ECVs for the remaining isolates in both CLSI and EUCAST. Nineteen VMDs were found including 12 *C. glabrata*, 6 *C. tropicalis* and one *C. krusei* isolate. For *C. glabrata* the CLSI and EUCAST ECV are the same, so the differences are clarified by the higher MICs produced by SY. Additionally, for *C. tropicalis,* SY had higher MICs. The same was the case for the *C. krusei* isolate, where the SY MIC was 1 mg/L versus 0.12 mg/L for M-AM with again the same ECV (0.5 mg/L) for CLSI and EUCAST. There was also one MD for a *C. guilliermondii* (SY MIC 0.25 mg/L (ECV 0.5 mg/L) versus M-AM MIC 0.5 mg/L (ECV 0.25 mg/L). 

The CA of voriconazole was based on the results of 66 *Candida* isolates because both CLSI and EUCAST, or in the case of *C. dubliniensis* CLSI alone, do not provide CBPs nor ECVs for 33 isolates. Twenty-two discordances were demonstrated, leading to a CA of 66.7%. These discordances were observed in 13 *C. glabrata* isolates, for which a big difference between the CLSI ECV and the EUCAST ECV is noticed, 0.25 versus 1 mg/L respectively, resulting in 13 VMDs. One *C. krusei* isolate had a MIC of 1 versus 0.12 mg/L (SY versus M-AM), which corresponded to an mD. The remaining mDs were found in six *C. tropicalis* and two *C. parapsilosis* isolates in various directions. CLSI and EUCAST have very similar CBPs for those species so these discordances were due to the difference in MIC value between SY and M-AM. 

Seventy-two *Candida* isolates were used to evaluate the CA of itraconazole. Twenty-seven *Candida* strains belonged to species for which no CLSI or EUCAST CBPs are available. Thirteen (18.1%) MDs were noted, of which 11 were *C. glabrata* isolates. The *C. glabrata* CLSI and EUCAST ECV of itraconazole is 4 versus 2 mg/L respectively. As a consequence, a MIC of 4 mg/L is assigned to another category according to the criteria used (*n* = 5). For the six other *C. glabrata* isolates, a MIC > 4 mg/L was found with the M-AM panel, while the SY MIC was ≤2 mg/L. One discordance was observed in a *C. lusitaniae* strain. A MIC of 0.5 versus 0.25 mg/L was obtained (SY versus M-AM), which was interpreted as WT versus non-WT. The last MD was seen in a *C. parapsilosis* strain (SY MIC 0.25 mg/L (ECV 0.5 mg/L) versus M-AM MIC 0.5 mg/L (R > 0.12 mg/L)).

For anidulafungin, 66 *Candida* isolates were used to calculate the CA, since no CLSI or EUCAST CBPs nor ECVs were available for 33 isolates. With 14 (21.2%) MDs and two (3.0%) mDs, anidulafungin had a CA of 75.8%. The 14 MDs comprised five *C. tropicalis*, five *C. glabrata*, two *C. albicans* and two *C. krusei* isolates. For *C. tropicalis* the CLSI CBPs are S ≤ 0.25, I = 0.5, R ≥ 1 mg/L versus the EUCAST CBPs S ≤ 0.064, R > 0.064 mg/L. Because of this distinct difference in breakpoints, common MIC results of 0.12 mg/L and 0.25 mg/L were categorized differently according to the criteria used. For four *C. glabrata* isolates the M-AM panel gave MIC values of 0.12 mg/L (interpreted as R according to EUCAST CBPs) while the corresponding SY MICs were 0.03 mg/L, 0.06 mg/L and in two cases 0.12 mg/L (all interpreted as S according to CLSI CBPs). For two *C. albicans* isolates, a MIC of 0.06 mg/L was obtained with the M-AM panel (categorized as R) with a corresponding SY MIC value of 0.12 mg/L (categorized as S). For two *C. krusei* isolates, an M-AM MIC of 0.25 mg/L was obtained (assigned as R) where the corresponding SY MIC values were 0.12 mg/L and 0.25 mg/L (both belonging to the S category). The two mDs were caused by two *C. parapsilosis* isolates. The SY MIC was 4 mg/L for both isolates which made them I according to CLSI. The M-AM MICs were 0.5 and 1 mg/L which attributed them to the S category according to EUCAST. 

For micafungin, 66 *Candida* isolates could be included. CLSI and EUCAST do not provide CBPs nor ECVs for 33 isolates. A CA of 93.9% was achieved. Two mDs were found for *C. parapsilosis*. For the two isolates, an SY MIC of 4 mg/L was found and a M-AM MIC of 0.25 and 0.5 mg/L (resulting in I versus S). Two MDs were seen in *C. albicans* isolates. The M-AM MIC of 0.03 mg/L is classified as R whereas the corresponding SY MIC was 0.015 mg/L in both strains (categorized as S). 

### 3.5. Comparison of Susceptibility Ratios

**Figure 2:**
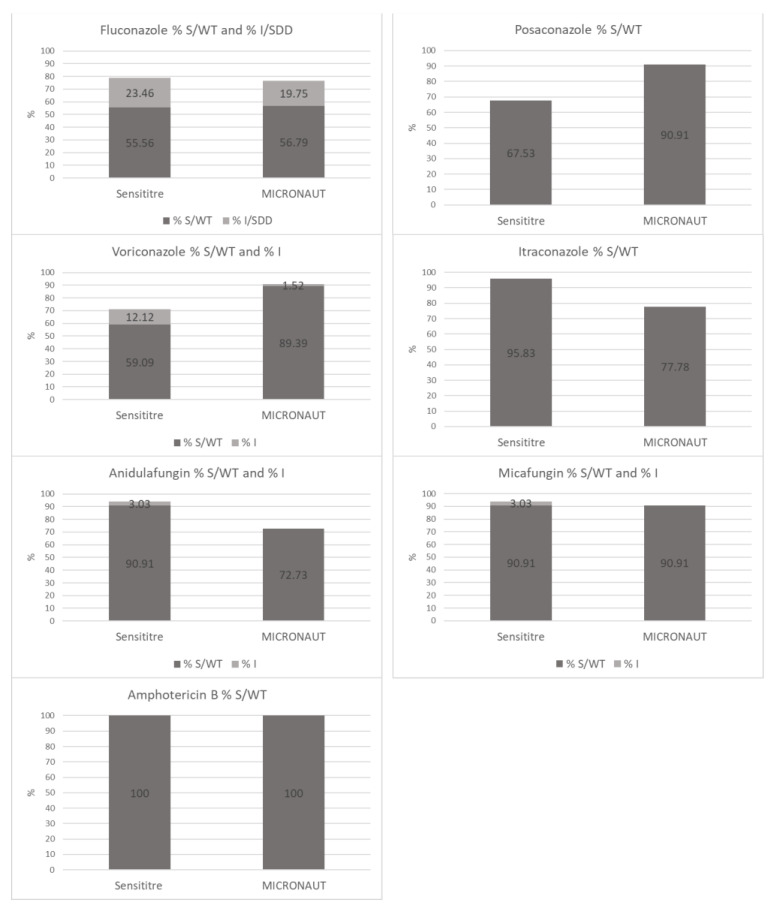
Comparison of SY versus M-AM: percentage of S/WT and I/SDD *Candida* isolates for each antifungal agent.

The percentage of S/WT and I/SDD *Candida* isolates for each antifungal agent was calculated with the M-AM system and the SY system (Figure 2). These figures reflect the consequences of a laboratory choice for SY or M-AM in daily clinical practice. For amphotericin B (100% vs. 100%), fluconazole (79.0% vs. 76.5%) and micafungin (93.9% vs. 90.9%) the susceptibility ratios (including S, WT, I and SDD results) are not statistically significantly different (SY vs. M-AM respectively). 

For posaconazole (67.5% vs. 90.9%; *p* < 0.05) and voriconazole (71.2% vs. 90.9%; *p* < 0.05), the susceptibility ratios were significantly higher using the M-AM system. 

In contrast, the susceptibility ratios for itraconazole (95.8% vs. 77.8%; *p* < 0.05) and anidulafungin (93.9% vs. 72.7%; *p* < 0.05) were significantly higher using SY.

## 4. Discussion

The aim of this study was to compare two commercial colorimetric broth microdilution antifungal susceptibility assays, SY and M-AM. 

First, the MIC values were compared as such and the EA for each antifungal agent was evaluated. The *C. lambica* isolate did not grow in both panels. Espinel-Ingroff et al. had the same observation. In their study, five out of seven *C. lambica* isolates did not grow (96 h) in either the YeastOne panel or in the NCCLS microdilution trays [28]. As there were no MIC values available for the *C. lambica* isolate, this strain was left out of the EA and CA calculations. As in several other studies, discrepancies among MIC endpoints of more than two dilutions were used to calculate the EA [8,10,11,29,30]. 

Pfaller et al. described that MIC results of the CLSI reference method tended to be one or more dilutions higher than those obtained with the EUCAST reference method for most antifungal agents and species [10]. This finding could partly be confirmed in our study with the commercial counterparts of these reference methods as for most antifungal agents the SY MICs were generally higher than the M-AM MICs (Figure 1). However, for itraconazole, the M-AM MICs were clearly higher than those of SY. Pfaller et al. also found that the CLSI MIC results for caspofungin tended to be one or two doubling dilutions lower than EUCAST MIC results [10]. In our study, the SY MICs for caspofungin tended to be higher than those obtained with M-AM (Figure 1).

Secondly, the CA for each antifungal agent was evaluated. Since the EA of fluconazole MICs was very high (98.0%), and since CBPs of CLSI and EUCAST are identical, the CA was also high (95.1%). CAs of other azoles, i.e., posaconazole (74.0%), voriconazole (66.7%) and itraconazole (81.9%), were rather low. The susceptibility ratios show statistically significant differences with a much larger proportion of the isolates considered to be S to posaconazole and voriconazole when using the M-AM system while on the contrary, itraconazole susceptibility ratio being much lower. Although there are some differences between EUCAST and CLSI CBP and ECVs, the MIC plots indicate that these differences are mainly due to the systematically higher MICs for posaconazole and voriconazole when using the SY system. The same is true for the higher MICs for itraconazole when the M-AM system is used (Figure 2). 

EUCAST states that isolates that are S to both anidulafungin and micafungin should be considered as S to caspofungin [26]. EUCAST does not provide a solution for conflicting results between anidulafungin and micafungin. CLSI describes that in vitro caspofungin susceptibility testing has been associated with significant interlaboratory variability. But in contrast to EUCAST, CLSI does provide caspofungin CBPs for the most common *Candida* species and S results for caspofungin may be reported as such. I or R results should be confirmed by additional susceptibility testing with anidulafungin or micafungin, by DNA sequence analysis of *FKS* genes or by sending the isolate to a referral laboratory for confirmation. *Candida* isolates R to anidulafungin or micafungin, or possessing characteristic *FKS* hot spot mutations are to be considered R to all echinocandins [24]. Like EUCAST, CLSI does not explain what to do when there is a S/I discordance between anidulafungin and micafungin. Whereas the high-level R proportion to micafungin is comparable and small (<10%) with both systems, there is a major difference in the susceptibility ratio (including full susceptible and intermediate susceptible isolates) for anidulafungin between both systems. The M-AM system (EUCAST criteria) categorizes 27.3% of all isolates considered as fully R, making anidulafungin or caspofungin an unacceptable choice for treatment. On the other hand, this R proportion is only 6.1% when the SY system is used (CLSI criteria). Since echinocandins are considered as first-choice therapy [31] for *Candida* bloodstream infections, the choice for implementation of one of these commercial systems could have a major impact on clinical practice, antifungal drug consumptions and possibly even on patient outcome. Amphotericin B and 5-flucytosine showed high EAs (100% and 94.9% (Table 3)). This resulted in an excellent CA for amphotericin B of 100%. However, it should be noted that no R or non-WT isolates were included.

In this study, it was not possible to evaluate which colorimetric broth microdilution method was more correct because SY is evaluated against the CLSI reference method, while M-AM is evaluated against the EUCAST reference method. The important statistically significant differences in test results between both systems do however expose a significant impact on local antifungal susceptibility patterns, on antifungal use and even on in-hospital empirical guidelines when one or the other commercial system is implemented in the clinical laboratory. Further research is warranted to clarify the important discordances on various levels. These studies should include data of resistance-conferring mutations for each isolate and/or clinical evaluation of patients treated with antifungals showing discordant susceptibility results in both commercial systems. 

## Figures and Tables

**Table 1:** Breakpoint table with CLSI and EUCAST CBPs and ECVs.

Antifungal Agent	Species	CLSI MIC CBP (mg/L)	CLSI ECV (mg/L)	EUCAST MIC CBP (mg/L)	EUCAST ECV (mg/L)
Fluconazole	*C. glabrata*	SDD ≤ 32, R ≥ 64	-	S ≤ 0.001, R > 16	-
	*C. albicans*	S ≤ 2, SDD 4, R ≥ 8	-	S ≤ 2, R > 4	-
	*C. parapsilosis*	S ≤ 2, SDD 4, R ≥ 8	-	S ≤ 2, R > 4	-
	*C. tropicalis*	S ≤ 2, SDD 4, R ≥ 8	-	S ≤ 2, R > 4	-
	*C. lusitaniae*	-	1	-	-
	*C. dubliniensis*	-	0.5	S ≤ 2, R > 4	-
	*C. guilliermondii*	-	8	IE	16
	*C. kefyr*	-	1	-	[1]
	Non-species related	-	-	S ≤ 2, R > 4	-
Posaconazole	*C. glabrata*	-	1	IE	1
	*C. albicans*	-	0.06	S ≤ 0.06, R > 0.06	-
	*C. parapsilosis*	-	0.25	S ≤ 0.06, R > 0.06	-
	*C. tropicalis*	-	0.12	S ≤ 0.06, R > 0.06	-
	*C. lusitaniae*	-	0.06	-	-
	*C. dubliniensis*	-	0.12	S ≤ 0.06, R > 0.06	-
	*C. krusei*	-	0.5	IE	0.5
	*C. guilliermondii*	-	0.5	IE	0.25
	Non-species related	-	-	IE	-
Voriconazole	*C. glabrata*	-	0.25	IE	1
	*C. albicans*	S ≤ 0.12, I 0.25–0.5, R ≥ 1	-	S ≤ 0.06, R > 0.25	-
	*C. parapsilosis*	S ≤ 0.12, I 0.25–0.5, R ≥ 1	-	S ≤ 0.125, R > 0.25	-
	*C. tropicalis*	S ≤ 0.12, I 0.25–0.5, R ≥ 1	-	S ≤ 0.125, R > 0.25	-
	*C. lusitaniae*	-	-	-	-
	*C. dubliniensis*	-	-	S ≤ 0.06, R > 0.25	-
	*C. krusei*	S ≤ 0.5, I 1, R ≥ 2	-	IE	1
	*C. guilliermondii*	-	-	IE	-
	Non-species related	-	-	IE	-
Itraconazole	*C. glabrata*	-	4	IE	2
	*C. albicans*	-	-	S ≤ 0.06, R > 0.06	-
	*C. parapsilosis*	-	0.5	S ≤ 0.125, R > 0.125	-
	*C. tropicalis*	-	0.5	S ≤ 0.125, R > 0.125	-
	*C. lusitaniae*	-	1	-	0.125
	*C. dubliniensis*	-	0.25	S ≤ 0.06, R > 0.06	-
	*C. krusei*	-	1	IE	1
	*C. guilliermondii*	-	2	IE	2
	Non-species related	-	-	IE	-
Anidulafungin	*C. glabrata*	S ≤ 0.12, I 0.25, R ≥ 0.5	-	S ≤ 0.06, R > 0.06	-
	*C. albicans*	S ≤ 0.25, I 0.5, R ≥ 1	-	S ≤ 0.03, R > 0.03	-
	*C. parapsilosis*	S ≤ 2, I 4, R ≥ 8	-	S ≤ 4, R > 4	-
	*C. tropicalis*	S ≤ 0.25, I 0.5, R ≥ 1	-	S ≤ 0.06, R > 0.06	-
	*C. lusitaniae*	-	1	-	-
	*C. dubliniensis*	-	0.12	-	-
	*C. krusei*	S ≤ 0.25, I 0.5, R ≥ 1	-	S ≤ 0.06, R > 0.06	-
	*C. guilliermondii*	S ≤ 2, I 4, R ≥ 8	-	IE	-
	Non-species related	-	-	IE	-
Micafungin	*C. glabrata*	S ≤ 0.06, I 0.12, R ≥ 0.25	-	S ≤ 0.03, R > 0.03	-
	*C. albicans*	S ≤ 0.25, I 0.5, R ≥ 1	-	S ≤ 0.016, R > 0.016	-
	*C. parapsilosis*	S ≤ 2, I 4, R ≥ 8	-	S ≤ 2, R > 2	-
	*C. tropicalis*	S ≤ 0.25, I 0.5, R ≥ 1	-	IE	0.06
	*C. lusitaniae*	-	0.5	-	-
	*C. dubliniensis*	-	0.12	-	-
	*C. krusei*	S ≤ 0.25, I 0.5, R ≥ 1	-	IE	0.25
	*C. guilliermondii*	S ≤ 2, I 4, R ≥ 8	-	IE	-
	Non-species related	-	-	IE	-
Caspofungin	*C. glabrata*	S ≤ 0.12, I 0.25, R ≥ 0.5	-	†	†
	*C. albicans*	S ≤ 0.25, I 0.5, R ≥ 1	-	†	†
	*C. parapsilosis*	S ≤ 2, I 4, R ≥ 8	-	†	†
	*C. tropicalis*	S ≤ 0.25, I 0.5, R ≥ 1	-	†	†
	*C. lusitaniae*	-	1	†	†
	*C. dubliniensis*	-	-	†	†
	*C. krusei*	S ≤ 0.25, I 0.5, R ≥ 1	-	†	†
	*C. guilliermondii*	S ≤ 2, I 4, R ≥ 8	-	†	†
	Non-species related	-	-	†	†
Amphotericin B	*C. glabrata*	-	2	S ≤ 1, R > 1	-
	*C. albicans*	-	2	S ≤ 1, R > 1	-
	*C. parapsilosis*	-	1	S ≤ 1, R > 1	-
	*C. tropicalis*	-	2	S ≤ 1, R > 1	-
	*C. lusitaniae*	-	2	-	[0.5]
	*C. dubliniensis*	-	0.5	S ≤ 1, R > 1	-
	*C. krusei*	-	2	S ≤ 1, R > 1	-
	*C. guilliermondii*	-	2	IE	[0.5]
	*C. kefyr*	-	2		[1]
	Non-species related	-	-	IE	-

ECVs indicated in brackets [] are tentative; IE: insufficient evidence that the species is a good target for therapy with the antifungal agent. † no EUCAST breakpoints available for caspofungin due to significant inter-laboratory variation in MIC ranges. Interpretation must be based on the anidulafungin and micafungin breakpoints.

**Table 2:** The intralaboratory reproducibility for SY and M-AM.

QC Strain	Antifungal Agent	Sensititre Modal MIC (mg/L)	MICRONAUT Modal MIC (mg/L)	Sensititre Percentage of MICs within Mode ± 1 Dilution	MICRONAUT Percentage of MICs within Mode ± 1 Dilution
	Fluconazole	1	1	100%	100%
	Posaconazole	0.06	≤0.008	100%	100%
	Voriconazole	0.015	≤0.008	100%	100%
	Itraconazole	0.12	≤0.03	100%	100%
ATCC 22019	Anidulafungin	1	0.25	100%	100%
*C. parapsilosis*	Micafungin	1	0.12	100%	100%
	Caspofungin	0.5	0.12–0.25	100%	100%
	Amphotericin B	0.5	0.25	100%	100%
	5-Flucytosine	0.5	≤0.06	100%	100%
	Fluconazole	32–64	32	100%	100%
	Posaconazole	0.25	0.03	100%	100%
	Voriconazole	0.25	0.03	100%	100%
	Itraconazole	0.25	≤0.03	100%	100%
ATCC 6258	Anidulafungin	0.12	0.03	100%	100%
*C. krusei*	Micafungin	0.12–0.25	0.06	100%	100%
	Caspofungin	0.5	0.25	100%	100%
	Amphotericin B	1	0.5	100%	100%
	5-Flucytosine	16	8	100%	75%

**Table 3:** The EA as the percentage of MICs within two twofold dilutions, the CA and the number and percentage CA discrepancies between SY and M-AM.

Antifungal Agent	EA (%)	CA (%)	Number and Percentage CA Discrepancies		
			mD	MD	VMD
Fluconazole	98.0	95.1	3 (3.7%)	0	1 (1.2%)
Posaconazole	38.3	74.0	0	1 (1.3%)	19 (24.7%)
Voriconazole	64.6	66.7	9 (13.6%)	0	13 (19.7%)
Itraconazole	72.7	81.9	0	13 (18.1%)	0
Anidulafungin	89.9	75.8	2 (3.0%)	14 (21.2%)	0
Micafungin	84.8	93.9	2 (3.0%)	2 (3.0%)	0
Caspofungin	94.9	-	-	-	-
Amphotericin B	100	100	0	0	0
*5-Flucytosine*	*94.9*	*-*	*-*	*-*	*-*

mD: minor discrepancy; MD: major discrepancy; VMD: very major discrepancy.
